# Facilitators and Barriers for Keeping Cool in an Urban Heat Island: Perspectives from Residents of an Environmental Justice Community

**DOI:** 10.1089/env.2022.0019

**Published:** 2023-11-30

**Authors:** Alina M. McIntyre, Madeleine K. Scammell, Maria Pilar Botana Martinez, Leila Heidari, Abgel Negassa, Roseann Bongiovanni, M. Patricia Fabian

**Affiliations:** Alina M. McIntyre is a doctoral student at the Department of Environmental Health, Boston University School of Public Health in Boston, Massachusetts, USA.; Dr. Madeleine K. Scammell is an Associate Professor at the Department of Environmental Health, Boston University School of Public Health in Boston, Massachusetts, USA.; Maria Pilar Botana Martinez is a doctoral student at the Department of Environmental Health, Boston University School of Public Health in Boston, Massachusetts, USA.; Leila Heidari is a doctoral candidate at the Department of Environmental Health, Boston University School of Public Health in Boston, Massachusetts, USA.; Abgel Negassa is a Research Assistant at the Department of Environmental Health, Boston University School of Public Health in Boston, Massachusetts, USA.; Roseann Bongiovanni is an Executive Director at the GreenRoots, Inc. in Chelsea, Massachusetts, USA.; Dr. M. Patricia Fabian is an Associate Professor at the Department of Environmental Health, Boston University School of Public Health in Boston, Massachusetts, USA.

**Keywords:** community-based, environmental justice, urban heat island, qualitative methods, interviews

## Abstract

**Background:**

Extreme heat is a leading cause of morbidity and mortality during summer months in the United States. Risk of heat exposure and associated health outcomes are disproportionately experienced by people with lower incomes, people of color, and/or immigrant populations.

**Methods:**

As qualitative research on the experiences of residents in heat islands is limited, this community-based study examined barriers and coping strategies for keeping cool among residents of Chelsea and East Boston, Massachusetts—environmental justice (EJ) areas that experience the urban heat island effect—through semistructured interviews and qualitative content analysis.

**Results:**

Results indicate that all participants (*n* = 12) had air conditioning, but high energy bills contributed to low use. Eight participants were self-described heat-sensitive, with five experiencing poor health in heat. In addition, nine reported insufficient hydration due to work schedules, distaste of water, or perceptions of it being unsafe.

**Discussion:**

This research highlights the importance of understanding perceptions of residents in EJ communities to contextualize vulnerability and identify multipronged heat coping strategies and targeted interventions.

## BACKGROUND

In the past 20 years, extreme heat events have increased in frequency and duration, affecting millions of people globally.^1^ Urban areas experience higher temperatures than rural areas due to greater impervious surface area, low tree cover, and highly concentrated fossil fuel use.^2^ These factors contribute to the urban heat island (UHI) effect, with urban daytime temperatures measuring 1°F–6°F higher than surrounding rural areas.^2,^^3^ Higher temperatures directly affect health, contributing to heat-related deaths, and illnesses such as heat exhaustion, heat stroke, or respiratory illnesses.^1^ During summer months, extreme heat is the leading cause of morbidity in the United States.^3^ However, summer UHI intensity across major U.S. cities disproportionately impacts people with lower incomes and people of color.^4^ In addition, non-U.S. citizens face 3.4 times the risk of heat-related deaths compared with U.S citizens, with Hispanic non-U.S. citizens having an even higher risk.^5^

Adapting to extreme heat involves technological and behavioral solutions. Many cities have mobilized research to address at-risk populations and reduce UHI impacts through nonprofit and grassroots organizations such as Groundwork USA and West Harlem Environmental Action.^6^^,^^7^ However, implementing appropriate and sustainable interventions requires an understanding of the knowledge, attitudes, and practices of UHI residents, in addition to structural constraints and historical factors that mediate those practices.^8^^,^^9^ Additional research is needed to explore personal experiences with heat, determinants of exposure risk, and mitigation strategies. This study leveraged a strong academic-community partnership to better understand heat coping strategies in an environmental justice (EJ) community.

### The Chelsea and East Boston Heat Study

The Chelsea and East Boston Heat Study (C-HEAT) is a partnership between investigators at Boston University School of Public Health, in Boston, MA, USA, and GreenRoots, Inc., a grassroots EJ organization in the neighboring city of Chelsea, MA. The groups established an advisory team: staff from Boston and Chelsea city governments and stakeholders from the housing authority, community development corporations, and regional nonprofits focused on heat resilience.

The city of Chelsea and neighborhood of East Boston include 45,000–55,000 residents, with 67% and 58%, respectively, identifying as Hispanic or Latinx.^10^^,^^11^ Approximately 45% of Chelsea residents identify as foreign-born, whereas 50% of East Boston's residents self-identify in this category.^10,11^ A significant percentage of residents in both areas live below the federal poverty level; 24% of Chelsea's residents compared with the state's 10.5%.^10^ These characteristics meet the demographic criteria of the Massachusetts Executive Office of Energy and Environmental Affairs, establishing census tracts as EJ populations.^12^ These areas also experience the UHI effect.^10,^^13^ EJ communities have historically been excluded from environmental health decision making and face structural inequities such as old housing stock, high tenant occupied multifamily buildings, high residential instability, poor air quality, economic pressures, immigration-related stressors, and high rates of violence.^13^

### Conceptual framework

The aim of C-HEAT is to build response capacity to extreme heat events for residents in Chelsea and East Boston. Partners and key stakeholders, including the foundation that funded C-HEAT, were committed to collective action and empowerment of each individual and agency partner, using a community-based participatory research approach as a guide.^14^ Specifically, we are a partnership between university members and community organizers in a grassroots EJ organization.

We work together on every step of the research: articulating the research questions, study design, collection of data, and interpretation of results.^14^ The study is ongoing, including a variety of data collection approaches that engage residents, policy makers, government agencies, and academics to ensure the voices and concerns of residents inform decision-making and research agendas.^14^ In the analysis reported herein, we sought resident perceptions of extreme heat and thermal comfort to gain understanding of social, structural, physical, and cultural barriers and opportunities for coping with extreme heat.

## METHODS

### Participants

An initial C-HEAT study recruited Chelsea and East Boston residents for an outdoor and in-home temperature monitoring campaign during June 2020. Invitations and recruitment materials were distributed by GreenRoots through e-mail lists, and flyers in laundromats, grocery stores, and bus stops. Criteria for inclusion were being at least 18 years of age, owning a smartphone, English or Spanish language proficiency, residing in Chelsea or East Boston for at least 1 year, and planning to live at their current residence at least 6 months. Quantitative surveys were conducted with participants on their use of air conditioning (AC), knowledge and use of community resources, employment, financial and physical health, and general demographics.

GreenRoots and Boston University investigators meet weekly to discuss data collection, analyses, and C-HEAT related events and activities. The current qualitative study is a follow-up to the initial survey (*n* = 21) to further query the original cohort about contextual factors and perspectives related to heat coping (Table 1). For example, the survey responses indicated that all participants had AC but reported not using it frequently. We wanted to probe deeper, asking more open-ended questions, to gain a better understanding of cooling behaviors and barriers. Semistructured interviews were conducted with 12 original C-HEAT participants in summer 2021. All participants consented to the secure recording and storage of interview data. The questionnaire and methodology for this study was approved by the Institutional Review Board of Boston University Medical Campus.

**Table 1.** Participant Characteristics, 2020 (Initial Survey Study, *n* = 21) and 2021 (Current Interview Study, *n* = 12)

Variable	2020,* n *(%)	2021,* n *(%)
City		
Chelsea	15 (68)	10 (83)
East Boston	7 (32)	2 (17)
Gender		
Female	16 (73)	9 (75)
Male	6 (27)	3 (25)
Preferred language spoken (Spanish/English)		
Spanish	12 (55)	5 (42)
English	10 (45	7 (58)
Foreign-born^a^		
Foreign-born	11 (50)	6 (55)
Race/ethnicity^a^		
Hispanic/Latinx	9 (41)	9 (82)
Non-Hispanic White	6 (27)	1 (10)
Other	4 (18)	1 (10)
Unknown or not reported	3 (14)	1 (10)
Age^a^		
Median (range)	50 (22–78)	45 (23–66)
Rent/own		
Rent	17 (77)	10 (83)
Own	4 (18)	2 (17)
Other	1 (5)	0 (0)
Income^a^		
<$50,000	13 (59)	5 (45)
>$50,000	9 (41)	6 (55)
Refused/Don't know	2 (9)	1 (10)
Employment status^a^		
Employed	13 (59)	10 (91)
Unemployed	9 (41)	1 (9)
Education^a^		
Less than high school diploma	3 (14)	1 (10)
High school diploma or GED	10 (45)	5 (45)
Higher than high school degree	9 (41)	5 (45)
Home inhabitants		
Adults (median, range)	2 (1–6)	3 (1–6)
Children (median, range)	1 (0–4)	1 (0–4)
Years lived in home		
<1 Year	3 (12.5)	0 (0)
1–2 Years	3 (12.5)	2 (17)
3–5 Years	6 (25)	4 (33)
>5 Years	12 (50)	6 (50)
AC type		
Central	5 (23)	2 (17)
Other (portable/wall/window)	17 (77)	10 (83)
Housing type		
Multifamily	16 (73)	10 (83)
Single family	4 (18)	2 (17)
No. of stories in home		
1	2 (9)	0 (0)
2	17 (77)	3 (25)
3	1 (5)	6 (50)
4+	0 (0)	4 (33)

^a^ One participant did not complete question in 2021 interview.

AC, air conditioning.

### Data collection and measures

Interviews were conducted through Zoom with a trained interviewer and note-taker. Note-takers recorded participant verbal responses and noted nonverbal gestures, affects, and emphases. We stored recorded Zoom sessions and notes in an IRB-approved secure drive available to research team members only. Seven interviews were conducted in English and five in Spanish. Interviews lasted ∼1 hour.

Semistructured primarily open-ended interview questions were designed using responses to our 2020 survey questions as the starting place to further explore reasons for participant heat-response behaviors. We sought to collect qualitative data in the domains of AC use, heat adaptation behaviors, and participant demographics (see Supplementary Data). Probes and follow-up questions were used to capture additional details.

### Analysis

**FIG. 1.**
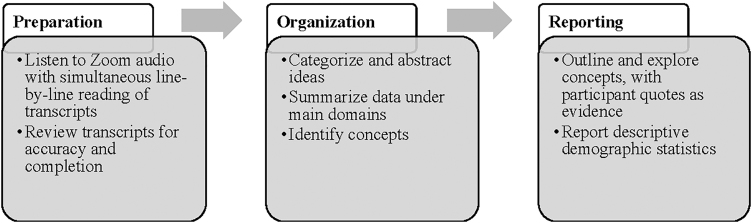
Directed qualitative content analysis process.

Qualitative analysis was guided by the three stages of directed content analysis: preparation, organization, and reporting (Fig. 1).^15^ First, interview transcripts were prepared and cross-checked with Zoom recordings for accuracy. All transcripts were read line-by-line two to three times to ensure comprehension. Spanish transcripts were translated into English by two research study team members who are native Spanish speakers. The organizational stage included categorization and abstraction of interview data.^16^

We created a summarization template in Excel, and interview data were organized within the following main topics (“domains”): heat coping facilitators, heat coping barriers, researcher interpretations, and relevant implications/actions. Exemplary participant quotes were included in the matrix next to each domain and participants were assigned pseudonyms. Team members collaborated on the identification of emerging concepts related to each domain. The reporting stage followed; our concepts, illustrated with quotes, were explained by data in each analytic domain. Progression through stages is shown in Figure 1. Descriptive statistics were used to analyze participant demographics.

## RESULTS

Eleven participants completed interviews in 2021, whereas one participant ended the interview approximately halfway through due to poor internet and competing demands. Most participants were renters in Chelsea, MA, female, and Hispanic/Latinx. Approximately half were foreign-born. Ages ranged from 23 to 66 years. The majority lived in multifamily, two- or three-story homes (“triple deckers”) common in these neighborhoods, and approximately half had lived more than 5 years in their home. All but one participant was employed (Table 1).

The main concepts that emerged from analyses of facilitators and barriers to coping with heat included the necessity of multipronged coping strategies (i.e., no single solution), the financial drain of AC use (i.e., keeping cool has its cost), health conditions exacerbated by heat, and barriers to proper hydration. Quotes that are translated from Spanish are indicated next to their pseudonym.

### No single solution for coping with heat

When asked how participants cool down when it is hot outside, only one person responded with a single solution. Mateo said, “I go to my room and turn on the AC.” All other participants described a series of actions: Turning on the AC, removing clothing, showering, opening windows and doors, turning on the fan (even if the windows are closed), and closing blinds or curtains.

I put on the AC, change my clothing, and close the curtains. (Ana)

I take off my clothes, I create a nest of towels where I'm sitting so that all of my sweat will be absorbed by the towels and not the couch, and sometimes I will splash cold water on my face. Now that the library is open I anticipate going to the library if it gets really bad. (Tom)

I drink some ice water, take a bath, and wear comfortable clothes. (Elena, translated)

When asked, all participants reported having window shades, and eight believed they effectively reduce heat. Eight participants also reported fan use, when prompted. Olivia, who owns her two-family home in Chelsea, uses window and portable AC units in combination with window shades:

We ended up purchasing shades for the windows…it really did end up mitigating solar heat gain into the office, living area…having shades is one of the most effective ways to deflect solar peaking. (Olivia)

While turning on AC was among the most frequent responses to our initial question, it was also described by Olivia and Paula as a “last resort” and “last option.”

### Keeping cool has its cost

All participants reported functioning AC. Two had central AC, and 10 had one or more window, wall, and/or portable units. Four participants were dissatisfied with their AC's ability to cool their home. The same four participants also expressed financial stress associated with AC use. In total, eight participants referred to the cost of AC as a barrier for its use:

If the central air does not work, you have to put in window units…then the bill goes up a lot. And then you have to choose to be hot or pay a lot more money…double the bill for AC. (Paula, translated)

AC does a really good job of digging into those electric bills. (Daniela)

Ana described concerns with overloading the capacity of her buildings' power supply:

When we have the high cool setting [on the AC], my light dims…I don't want to cause any power outage in my apartment…it's really hard to pay [the energy bills].

Seven participants remarked on the cost, age, and installing difficulties of their AC units as disincentives for use:

They are old devices, used. …Buying one [new] is much more expensive…maybe because they are old they use up more energy. (Paula, translated)

There is nowhere to put it…the window units are very expensive. (Rosa, translated)

### Heat impairs health

Eight participants reported that they were especially sensitive to heat, with five mentioning health problems that are exacerbated when it is hot. These included skin conditions (rashes and eczema), asthma, heat exhaustion, and menopause:

I'm very susceptible to heat stroke, really susceptible…I've suffered from an episode about every summer…and then the nausea, vomiting, intense headache that is blinding. My body doesn't seem like it is able to regulate heat like it used to. (Daniela)

Ana, who has eczema and asthma, mentioned her increased symptoms in the heat:

I make sure to have my lotions, steroid creams, all stocked up…summer is more burdensome.… I remember I passed out because it was so hot. I was at a park with some of my cousins.

Of the self-described heat-sensitive participants, two reported working in environments that may make them more vulnerable. Rosa cleans homes and explained that some residents keep the AC off while she is cleaning:

The owners say that we are there to clean, not for comfort. (translated)

Maria works in a restaurant kitchen:

If it's 90 outside, it is more than 100 degrees inside…So we have to be drinking a lot of water…In the previous heat wave when I was working I got dizzy. (translated)

### Knowledge versus practice: barriers to hydration

Participants described awareness of many heat-related health concerns and prevention strategies, including the importance of proper hydration as reflected in Maria's aforementioned quote. However, of 12 participants, 9 felt that they did not drink enough water—consuming approximately five and a half cups of water per day, with a range of four to eight cups.

Two women (not including Maria) who both have demanding full-time jobs and consider themselves to be heat-sensitive, described the inconvenience of having to urinate as the reason for not drinking enough water:

Drinking so much water is going to make me pee, so I'm drinking less water. (Ana)

If I consume too much liquid, it's just not conducive to my workday. (Olivia)

Two participants also did not like the taste of water, and associated the taste with quality:

I've never liked the flavor…I've always had a hard time drinking it. They say it's not healthy to drink tap water so I don't drink it. My family will say “don't drink from the sink,” so I'm not drinking that… in Chelsea, and when I lived in Puerto Rico. (Sara)

Julian, who also lived in Puerto Rico before moving to Chelsea, uses a filter to clean his drinking water:

Last year the commissioner of DPW [Department of Public Works] was talking about how this water was coming from somewhere in Massachusetts that is clean [but] I am an over-thinker… What about the pipes? What about things getting contaminated…? (Julian)

Paula, who moved from Mexico to Chelsea between 5 and 10 years ago, echoed this suspicion:

The water quality is good, but I think it's the pipes [that are contaminated]. (translated)

Nine participants reported taking steps to clean their drinking water. Although seven of the nine indicated they felt that their drinking water is safe, eight use a filtration system and Carmen said she sometimes boiled her water so it would feel “more trustworthy.” Overall, four participants discussed perceived safety issues such as dangerous pipes and unknown contaminants.

## DISCUSSION

**FIG. 2.**
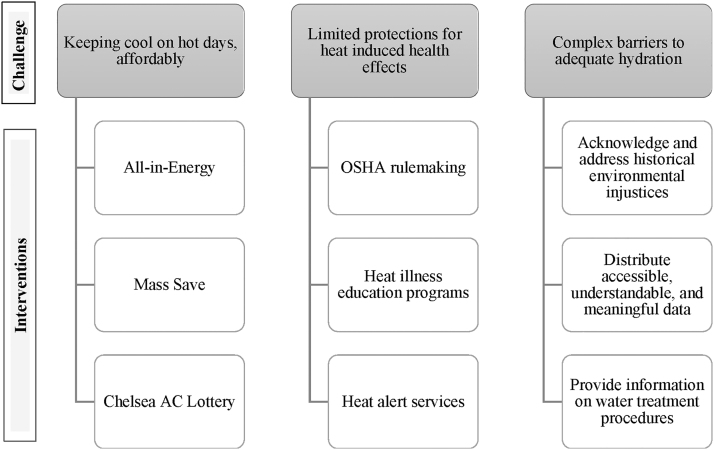
Challenges and select interventions for coping with heat.

Our interviews with residents of Chelsea and East Boston, MA, illuminate shared experiences of coping with heat, financial stress, health problems, and barriers to proper hydration. These challenges present opportunities for strengthening current interventions and developing new solutions (Fig. 2).

Using AC on hot days presented participants with a dilemma: thermal comfort and healthier conditions versus high energy bills. Similarly, a survey of Houston, Texas, residents found that people who experience discomfort and/or heat-related symptoms at home chose to reduce or forgo AC because of high electricity cost.^10^ In addition to research in Houston, other studies report a similar trade-off between cooling and high energy bills in vulnerable populations, and the importance of integrating energy efficiency and weatherization in sustained financial assistance.^16^^–^^18^

C-HEAT participants suggested that if cost were not a barrier, they would purchase newer more efficient AC units. In Massachusetts, heat adaptation interventions include reducing financial barriers to AC use. All-in-Energy promotes subsidized energy costs for low-income households, and Mass Save, a collaborative of MA utility servicers, offers small rebates for new window AC units.^19^^,^^20^ Additional states have implemented programs to offset energy bill and AC costs, including home weatherization assistance in Phoenix, Arizona, and the home energy assistance in New York.^21^^,^^22^

In summer 2021, Chelsea had its first AC lottery. With support from the Barr Foundation (also funder of C-HEAT), the city provided nearly 100 AC units to residents randomly selected from nearly 1000 applicants. Winning residents also received a check for $300 to offset the cost of electricity.^23^ Future research evaluating access and reach of these programs, and additional options such as cooling centers, may be beneficial when developing and refining heat adaptation interventions. Regulations at the MA State level should prioritize prevention of shutting off electricity during summer months for those who cannot pay their electric bills, as no such protection currently exists.^24^

C-HEAT participants mentioned asthma, eczema, and menopause as conditions that worsened with heat exposure. Research indicates that heat contributes to poor air quality and asthma symptoms, as it traps respiratory irritants at the ground level.^25^ Although there is minimal research assessing physiological symptoms of menopause and heat, studies show that hot flashes and increased heart rate may worsen with physical exertion, dehydration, and/or environmental heat exposure.^26^ Also, as people age, their hormones and thermoregulatory capacity change, reducing ability to dissipate heat.^27^^,^^28^

Participants also faced specific health challenges in their workplaces. Immigrant rights organizations including United Farmworker Foundation have advocated for heat stress standards. Presently, there are no Occupational Safety and Health Administration (OSHA) standards nor MA-specific laws that monitor heat exposure or prevent heat stress.^29^ However, in 2021, OSHA began the rulemaking process for “Heat Injury and Illness Prevention in Outdoor and Indoor Work Settings.”^29^ In addition, groups have developed heat illness education programs including heat safety initiatives using health fairs, block parties, promotores (community health workers), health information handouts, and bilingual websites with information on employee rights and guidelines.^30^

Although community-based interventions are limited in MA, the City of Boston offers a heat alert service and multilingual fact sheets for general residents and those with specific health conditions.^31^ As of 2020, the Center for Disease Control's Climate-Ready States and Cities Initiative has provided funding to 16 states (including MA) to build local capacity to address heat and implement health adaptation plans, providing a database to reference these cities' efforts in detail.^32^

Despite understanding benefits of proper hydration, many C-HEAT participants disliked or mistrusted their water. An American Housing Survey analysis found that perception of water quality is strongly associated with income and “minority status,” with one of the strongest associations being foreign-born from Latin America.^33^ A number of participants reported influence by their experiences in Central America and Puerto Rico.

However, mistrust of water safety may also stem from underlying historical environmental injustices faced by immigrants and communities of color in the United States. The Natural Resources Defense Council found that drinking water systems that violated the Safe Drinking Water Act were 40% more likely to occur in places with higher percentages of low-income non-native English speakers.^34^ Research suggests that emphasizing water treatment procedures, having accessible water quality data, and providing information on financial and environmental costs associated with plastic water bottles, versus tap water, may promote trust in drinking water sources and improve hydration practices.^35^

Although strategies have been used to reduce heat and associated financial and health challenges, further study is needed to understand and address the unique needs of populations residing in EJ neighborhoods such as Chelsea and East Boston. Findings from this analysis have been shared with advisory team members and will inform future interventions, evaluations, and research by academic investigators and our municipal partners. Study participants are engaged in a photovoice project and will be presenting the results of their analyses in summer 2022. Future research including larger numbers of individuals in a variety of urban EJ communities would help inform the extent of the challenges and where to direct policies and intervention resources.

### Study limitations

Owing to the COVID-19 pandemic, our capacity to recruit and engage participants was limited to technologically mediated contact. Semistructured interviews were conducted over Zoom, relying on strong internet connectivity and access to smartphones or computers. Our sample size would likely have been larger and more diverse had we been able to recruit and engage participants in person as originally intended. Finally, we acknowledge the potential for socially desirable participant responses due to the status and position of researchers versus community members.

### Contribution to the literature

Our study illuminates a variety of approaches used to cope with ambient heat in both a UHI and EJ community. We explore and highlight the trade-off experienced by residents facing thermal comfort and health versus high utility bills and financial stress. Participants also raised concerns regarding drinking water quality, and disincentives for proper hydration in the work setting where occupational exposures to heat may contribute to health issues. Overall, we identified barriers that can assist Chelsea and East Boston, along with other interested cities, when implementing heat adaptation and climate mitigation measures.

## Supplementary Material

Supplemental data

## Notes

^1^
World Health Organization. Heat and Health. <https://www.who.int/news-room/fact-sheets/detail/climate-change-heat-and-health>. (Last accessed on November 15, 2021).

^2^
Shushi Peng, Shilong Piao, Philippe Ciais, Pierre Friedlingstein, Catherine Ottle, François Marie Bréon, Huijuan Nan, Liming Zhou, and Ranga B. Myneni. “Surface Urban Heat Island across 419 Global Big Cities.” *Environmental Science and Technology* 46 (2012): 696–703.

^3^
Angel Hsu, Glenn Sheriff, Tirthankar Chakraborty, and Diego Manya. “Disproportionate Exposure to Urban Heat Island Intensity across Major US Cities.” *Nature Communications* 12 (2021): 1–11.

^4^
Jeremy Hoffman, Vivek Shandas, and Nicholas Pendleton. “The Effects of Historical Housing Policies on Resident Exposure to Intra-Urban Heat: A Study of 108 US Urban Areas.” *Climate* 8 (2020): 12.

^5^
Ethel Taylor, Ambarish Vaidyanathan, W. Dana Flanders, Matthew Murphy, Merianne Spencer, and Rebecca S. Noe. “Differences in Heat-Related Mortality by Citizenship Status: United States, 2005–2014.” *American Journal of Public Health* 108 (2018): S131–S136.

^6^
Groundwork USA. Groundwork USA. <https://groundworkusa.org/>. (Last accessed on November 15, 2021).

^7^
WE ACT for Environmental Justice. WE ACT for Environmental Justice. <https://www.weact.org/>. (Last accessed on December 10, 2021).

^8^
Olga Wilhelmi and Mary H. Hayden. “Connecting People and Place: A New Framework for Reducing Urban Vulnerability to Extreme Heat.” *Environmental Research Letters* 5 (2010): 014021.

^9^
Mary Hayden, Olga V Wilhelmi, Deborah Banerjee, Tamara Greasby, Jamie Cavanaugh, Vishnu Nepal, and Stephanie Gower. “Adaptive Capacity to Extreme Heat: Results from a Household Survey in Houston, Texas.” *Weather, Climate, and Society* 9 (2017): 787–799.

^10^
Healthy Chelsea. Who We Are: Our History. <https://healthychelsea.org/who-we-are/>. (Last accessed on December 10, 2021).

^11^
Boston Planning and Development Agency Research Division. Boston by the Numbers 2020. <http://www.bostonplans.org/research/research-publications-overview/boston-by-the-numbers>. (Last accessed on December 10, 2021). Boston Planning and Development Agency Research Division. Boston by the Numbers 2020. <http://www.bostonplans.org/research/research-publications-overview/boston-by-the-numbers>. (Last accessed on December 10, 2021).

^12^
Massachusetts Executive Office of Energy and Environmental Affairs. Environmental Justice Populations in Massachusetts. <https://www.mass.gov/info-details/environmental-justice-populations-in-massachusetts>. (Last accessed on April 5, 2022).

^13^
Urban Land Institute Boston/New England. Living with Heat. <https://ulidigitalmarketing.blob.core.windows.net/ulidcnc/2019/11/Living-With-Heat-Report-for-web.pdf>. (Last accessed on January 15, 2022).

^14^
Peggy M. Shepard, Mary E. Northridge, Swati Prakash, and Gabriel Stover. “Preface: Advancing Environmental Justice through Community-Based Participatory Research.” *Environmental Health Perspectives* 110 (2002): 139–140.

^15^
Sato Elo and Helvi Kyngäs. “The Qualitative Content Analysis Process.” *Journal of Advanced Nursing* 62 (2008): 107–115.

^16^
Jamal Lewis, Diana Hernández, and Arline T. Geronimus. “Energy Efficiency as Energy Justice: Addressing Racial Inequities through Investments in People and Places.” *Energy Efficiency* 13 (2020): 419–432.

^17^
Carol Farbotko and Gordon Waitt. “Residential Air-conditioning and Climate Change: Voices of the Vulnerable.” *Health Promotion Journal of Australia* 22 (2011): 13–15.

^18^
Enrica de Cian, Filippo Pavanello, Teresa Randazzo, Malcolm N. Mistry, and Marinella Davide. “Households' Adaptation in a Warming Climate. Air Conditioning and Thermal Insulation Choices.” *Environmental Science and Policy* 100 (2019): 136–157.

^19^
Mass Save. Cooling Equipment. <https://www.masssave.com/saving/equipment-guide>. (Last accessed on January 15, 2022).

^20^
All in Energy. All in energy: Income eligible programs. <https://allinenergy.org/income-eligible-programs.html>. (Last accessed on November 15, 2021).

^21^
Chrissie Bausch, Kristi Eustice, Alison Cook-Davis, Imani Cruz, Melina Cruz, Paige Riddle, Madison Frazee, Ellen O'Brien, and Erica Quintana. *Are State and Local Policies Addressing Community Concerns about Extreme Heat? A Case Study of American Indian and Latino Communities in Phoenix, Arizona*. (Phoenix, AZ: Arizona State University, 2021).

^22^
Office of Temporary and Disability Assistance, New York State. Cooling Assistance, Home Energy Assistance Program (HEAP). <https://otda.ny.gov/programs/heap/#cooling-assistance>. (Last accessed on January 15, 2022).

^23^
Martha Bebinger. Cooling aid shifts from luxury to necessity for some in Mass. WBUR. <https://www.wbur.org/news/2021/07/23/cooling-assistance-climate-change-massachusetts>. (Last accessed on November 15, 2021).

^24^
Attorney General's Consumer Advocacy & Response Division. When am I protected from having my utilities shut off? <https://www.mass.gov/service-details/when-am-i-protected-from-having-my-utilities-shut-off>. (Last accessed on April 15, 2022).

^25^
Alison Kenner, Alexandra Skula, Deepa Mankikar, Ian Zimmermann, Eliza Nobles, Julia Menzo, Thomas Flaherty, and Russell Zerbo. “The Climate-Ready Home: Teaching Climate Change in the Context of Asthma Management.” *Environmental Justice* 13 (2020): 101–108.

^26^
Josh Foster, Simon G. Hodder, Alex B. Lloyd, and George Havenith. “Individual Responses to Heat Stress: Implications for Hyperthermia and Physical Work Capacity.” *Frontiers in Physiology* 11 (2020): 1147.

^27^
Alison Millyard, Joe D. Layden, David B. Pyne, Andrew M. Edwards, and Saul R. Bloxham. “Impairments to Thermoregulation in the Elderly During Heat Exposure Events.” *Gerontology and Geriatric Medicine* 6 (2020): 1–9.

^28^
Nisha Charkoudian and Nina S. Stachenfeld. “Reproductive Hormone Influences on Thermoregulation in Women.” *Comprehensive Physiology* 4 (2014): 793–804.

^29^
United States Department of Labor, and Occupational Safety and Health Administration. “US Department of Labor Initiates Rulemaking to Protect workers, Outdoors and Indoors, from Heat Hazards Amid Rising Temperatures.” <https://www.osha.gov/news/newsreleases/national/10262021>. (Last accessed on January 15, 2022).

^30^
Labor Occupational Health Program University California, Berkeley. Heat Illness Prevention. <https://ehs.berkeley.edu/safety-subject/field-research/heat-illness-prevention-and-response>. (Last accessed on December 5, 2021).

^31^
City of Boston. Keeping cool in the heat. <https://www.boston.gov/departments/emergency-management/keeping-cool-heat>. (Last accessed on December 5, 2021).

^32^
Emmanuelle Hines, Climate and Health Program, and Centers for Disease Control and Prevention. *Extreme Heat Adaptation: How the Climate-Ready States and Cities Initiative (CRSCI) Grant Recipients Are Preparing for and Responding to Increasing Temperatures*. (2020).

^33^
Gregory Pierce and Silvia Gonzalez. “Mistrust at the Tap? Factors Contributing to Public Drinking Water (Mis) Perception across US Households.” *Water Policy* 19 (2017): 1–12.

^34^
Kristi Pullen Fedinick, Steve Taylor, and Michele Roberts. “Watered Down Justice.” *Natural Resources Defense Council* (2019): 1–52.

^35^
Sohyun Park, Stephen Onufrak, Aisha Patel, Joseph R Sharkey, and Heidi M Blanck. “Perceptions of Drinking Water Safety and Their Associations with Plain Water Intake among US Hispanic Adults.” *Journal of Water and Health* 17 (2019): 587–596.

