# Supplementary material for: Facilitators and Barriers for Keeping Cool in an Urban Heat Island: Perspectives from Residents of an Environmental Justice Community
Source: Environ Justice. 2023 Nov 30;16(6):410–7. doi: 10.1089/env.2022.0019 (PMC10704574; doi:10.1089/env.2022.0019)
Supplement: Supplemental data [file Supp_Data.docx]

Introduction:

Hello, my name is [insert name]. I am a member of the C-HEAT research team. Thank you again for taking the time to talk with me today.

As you know, our research is focused on heat, and you will participate in a photovoice project. Before we start the group discussions, however, we wanted to conduct one-on-one interviews with participants to get in-depth responses to questions about your own personal heat experience. …

I will ask questions but I hope this is like a discussion, and I hope it is enjoyable. Our research team will be reading these interviews and we may write about them. If we do, we will not use your names, so what you share with me will be confidential. I will refer to you by your first name only….

The interview will take about 1 hour.

Before we begin, I just want to ask you a few questions.

1. Do I have your permission to record the audio of our conversation today?
2. Do you have any questions?

OK, I’ll start by asking about **your home.**

- How long have you lived in your current home?
- Do you rent or own?
- How did you decide to live here? [find out what factors contributed to their decision- rent, location, building type, public housing, section 8, etc.]
  - - Probes:
    - A public housing resident living in a building owned by the Housing Authority?
    - A resident of a building managed by a program or organization that provides affordable housing. If so, which program? ____________________
    - Part of a household that receives rental assistance, such as a “Section 8” voucher
- Is it a single family home or multi-family? How many apartments in your building?
  - - How many floors are in your building?
    - On what floor(s) do you live?

Can you tell me about who else, if anyone, lives with you?

[get information on number of people, ages, relationships, etc.]

Now how much time do you spend at home, typically?

- About how much time do you spend in your home on a typical weekday? How about a weekend day?
- How much has where you usually spend your time changed since last summer? Why?

Thank you.

How would you describe typical living conditions in your residence during last SUMMER, in terms of how hot or cool it felt? (hot, warm, about right, cool, or cold)

- - What are some of the reasons you think that your home felt that way (hot, warm, about right, cool, or cold) last summer?
- Do you have air conditioning that works anywhere in your home?
  - If so, what kind(s) (window, wall, portable, central)?
  - In what room(s)?
  - If not, what are the reasons why not? (i.e. You don’t need it, It’s too expensive, You want to conserve electricity, Your building’s wiring is not equipped to run an AC, You don’t like air conditioning, Building owner does not allow)
- Where did you get your AC unit?
  - Do you remember when you got it? How old is it?
- How do you install your AC unit? Did you install it or did someone help you?
- Do you install and remove it seasonally? Why or why not?
- When are you most likely to use the AC?
  - How often do you use your air conditioning?
  - How often during a typical summer week?
  - How about during the hottest week?
  - Does your use differ between the day and night? How about weekdays vs weekends?
  - and at what setting? (a certain temperature value, high/low, etc.)
- Do you think your AC is effective? Does it keep you cool enough? Are you satisfied by it?

We would like to ask you questions about your (person being interviewed only) health. As a reminder, you do not have to answer any question you feel uncomfortable answering. I will start by asking about your overall health.

- How would you describe your overall health? Do you have any particular health concerns or conditions?
- Do you think you are especially sensitive to heat for any reason? If so, what reasons?)
  - Do you manage any health conditions you have differently when it is hot in the summer?
- How would you describe your overall sleep quality? How good is your sleep, and how much do you get?
- Are there specific things in your environment that disrupt your sleep (e.g., noise, temperature, neighbors, temperature)?
- On a typical day, how many 8oz glasses of liquid (show picture of 8oz glass) do you consume:
  - Coffee / Tea ___1__
  - Milk ______
  - Water _____
  - Juice _____
  - Alcohol ______
  - Soda _____
  - Other
- In general, do you feel that you drink enough water [circle response: Yes / no / Refused ]?
  - If no, why do you not drink enough water?
  - if yes, what helps facilitate that?
  - Do you know where the water that you drink comes from? If yes, where?
  - Do you think it is safe? If no, why not?

Adaptation

- Could you walk me through the steps you take to cool down when it is hot outside?
- What are the first things that you do?
- Do you have blinds or shades?
  - if so, how often do you close them? what keeps you from using them? do you use them to help with heat?
- Do you ever use a fan?
  - Window fan? Ceiling fan? Where? What room?
- Do you ever leave your house for a cooler area when your residence gets too hot?
  - If yes, where do you usually go?
  - If no, why don’t you leave home to find a cooler place during hot weather?
- How do you determine when it is hot enough for you to take action to cool down?

Heat wave education

- Have you ever been concerned that, on very hot days, the heat indoors could cause you to become ill? What about outside?
- Can you tell me any symptoms you might expect from too much heat?
  - have you ever experienced that? could you tell me about that?
- Does anything make you worried about others in your household or community getting sick because of the heat?
- Where have you seen, heard, or read information about dangerously hot weather in your community? **heat warning?**
  - What was the source of this information __________________
  - [If they say “news” ask what news source]
- What, if anything, do you typically do (or would you do) when you read or hear heat warning information?

Cooking

- How often do you cook at home? how about anyone else in your home?
- How often do you use exhaust fan or opening windows when cooking
- How long do you use the stove?
- Do you change how long you use the stove in hot weather?

Transportation

- What is your normal mode of transportation (to work, for groceries)?
- Do you change your routine when it is hot outside? How? How does this change your experience?
- If you use public transportation, do you usually go to an open station (i.e. unprotected bus /train station)?

General

- How would you describe your employment status?
  - if working: do you travel outside your home for work? how often?
- How would you describe your work schedule?

Which category represents the total combined income of all members of this household during the past 12 months? This includes money from jobs, net income from business, rent, pensions, social security payments and any other money income received (by members of this household who are 15 years of age or older.)

1. □ Less than $ 24,999
2. □ $25,000 to $49,999
3. □ $50,000 to $74,999
4. □ $75,000 to $99,999
5. □ $100,000 or more

99. □ Don’t Know

88. □ Refused to answer

How many household members are supported by your total combined household income (including yourself)?

How many of those people are children?

- Do you or someone in your household pay for cooling and heating at this residence, separate from your monthly rent or mortgage?
- At the present time, how easy or difficult is it to pay your bills?
- Do you sometimes have to make choices about which bills to pay during a given month?
  - If yes, If you have to make choices about which bills to pay, how do you prioritize
- Do you ever have difficulty paying heating or cooling bills?
- What year were you born? __________ (YYYY)

88. □ Refused to answer

- How do you identify your Gender? 1. □ Woman 2. □ Man 3. Gender Non-Binary 4. □ Other, specify: 3a. __________

99. □ Refused

- What is the highest degree or level of school you have completed (in US or elsewhere)?

| A | Less than high school diploma or GED | 1 □ |
| --- | --- | --- |
| B | High school diploma or GED | 2 □ |
| C | Some college but no degree | 3 □ |
| D | Associate degree | 4 □ |
| E | Bachelor’s degree (i.e. BA, BS) | 5 □ |
| F | Post graduate degree (i.e. masters or doctoral) | 6 □ |
| G | Refused to answer | 88 □ |
| H | Don’t know | 99 □ |

- Are you Hispanic or Latino/a?

| a | 1 □ Yes - Hispanic or Latino | A person of Cuban, Dominican, Mexican, Puerto Rican, Central American (ex. Belize, Guatemala, Honduras, El Salvador, Nicaragua, Costa Rica, Panama), South American (ex. Argentina, Brazil, Chile, Columbia, Ecuador, Peru, Venezuela), or other Spanish culture or Origin, regardless of race.  **If participants specifies from where, write in here:** __________ |
| --- | --- | --- |
| b | 2 □ No, Not Hispanic or Latino |  |
| c | 3 □ Other | Specify: cc. ____________________________ |
| d | 99. □ Don’t Know |  |
| e | ☐ Refuse to answer |  |

- What is your race? (*Select all that apply):*

| a | American Indian or Alaska Native | 1 □ | A person having origins in any of the original peoples of North, Central, or South America, and who maintains tribal affiliations or community attachment (ex. Cherokee, Chippewa, Navajo, Sioux Tribal Groups.) |
| --- | --- | --- | --- |
| b | Asian | 2 □ | A person having origins in any of the original peoples of the Far East, Southeast Asia, or the Indian subcontinent including, Cambodia, China, Hmong, India, Japan, Korea, Laotian, Malaysia, Pakistan, the Philippine Islands, Thailand, and Vietnam. |
| c | Black or African American | 3 □ | A person having origins in any of the black racial groups of Africa. (Including: Haitian, African Caribbean, Cape Verdean, African ancestry, or other.) |
| d | Native Hawaiian or Other Pacific Islander | 4 □ | A person having origins in any of the original peoples of Hawaii, Guam, Samoa, or other Pacific Islands. (ex. Chamorro, Fijian, or Tongan) |
| e | White | 5 □ | A person having origins in any of the original peoples of Europe, the Middle East, or North Africa. |
| f | Unknown/not reported | 6 □ |  |
| g | Other | 7 □ | Specify: gg. __________________________________ |
| h | Don’t Know | 99. □ |  |
| i | ☐ Refuse to answer |  |  |

- Were you born in the U.S.?

1. □ Yes
2. □ No
3. □ Refused to answer
4. □ Don’t know

**If No,** (born outside in US), how long have you lived in the U.S.?

1. □ >10 years
2. □ 5-10 years
3. □ 3-5 years, not including 5
4. □ 1-3 years, not including 3
5. □ Less than a year

99. □ Don’t know

☐ Refuse to answer

- Where did you live before Chelsea/East Boston, if anywhere, and how long ago?

1. □ United States, specify: 1a. _______________

1. □ Elsewhere in North America, specify: 1a. _______________

2. □ Central America, specify: 2a. _______________

3. □ South America, specify: 3a. _______________

4. □ Asia, specify: 4a. _______________

5. □ Indian subcontinent, specify: 5a. _______________

6. □ Europe, specify: 6a. _______________

7. □ Africa, specify: 7a. _______________

8. □ Caribbean, specify: 8a. _______________

9. □ Other, specify: 9a. _______________

99. □ Don’t know

☐ Refuse to answer
